# Reconstruction of a Large Anterior Ear Defect after Mohs Micrographic Surgery with a Cartilage Graft and Postauricular Revolving Door Flap

**DOI:** 10.1155/2015/484819

**Published:** 2015-09-03

**Authors:** Stephanie Nemir, Lindsey Hunter-Ellul, Vlad Codrea, Richard Wagner

**Affiliations:** ^1^Division of Plastic Surgery, Department of Surgery, University of Texas Medical Branch, Galveston, TX 77555, USA; ^2^Department of Dermatology, University of Texas Medical Branch, Galveston, TX 77555, USA; ^3^School of Medicine, University of Texas Medical Branch, Galveston, TX 77555, USA

## Abstract

A novel postauricular revolving door island flap and cartilage graft combination was employed to correct a large defect on the anterior ear of an 84-year-old man who underwent Mohs micrographic surgery for an antihelical squamous cell carcinoma. The defect measured 4.6 × 2.4 cm and spanned the antihelix, scapha, a small portion of the helix, and a large segment of underlying cartilage, with loss of structural integrity and anterior folding of the ear. The repair involved harvesting 1.5 cm^2^ of exposed cartilage from the scaphoid fossa and then sculpting and suturing it to the remnant of the antihelical cartilage in order to recreate the antihelical crura. The skin of the posterior auricle was then incised just below the helical rim and folded anteriorly to cover the cartilage graft. The flap remained attached by a central subcutaneous pedicle, and an island designed using the full-thickness defect as a stencil template was pulled through the cartilage window anteriorly to resurface the anterior ear. This case demonstrates the use of the revolving door flap for coverage of large central ear defects with loss of cartilaginous support and illustrates how cartilage grafts may be used in combination with the flap to improve ear contour after resection.

## 1. Introduction

The auricle is a very common site of skin cancers due to its projection from the head and subsequent actinic exposure [1]. This is particularly true in men, due to hairstyles that often do not protect the ear. Most auricular skin cancers are located on the helix, antihelix, and posterior ear, less frequently involving the lobule, tragus, or conchal bowl [2]. Large anterior auricular defects are a reconstructive challenge due to the complex topography of the ear, and second intention healing can lead to poor cosmetic outcomes, chondritis, or infection [3].

Repair is optimal for helical defects due to the high likelihood of cartilage desiccation and fibrosis that could result in notching [3]. One of the primary goals of ear reconstruction is to correct the shape of the auricle such that it does not attract attention when viewed by someone standing at a conversational distance [4]. Surgical options include second intention healing, skin grafting, preauricular transposition flap, tubed pedicle flap, postauricular pull-through flap, and postauricular revolving door island flap [3]. We describe a patient with a large defect of the anterior ear resulting in compromised structural integrity. The defect was repaired with a cartilage graft that restored the antihelical contour, followed by a postauricular revolving door (also known as a trap door or flip-flop) island flap that restored the ear's structural integrity and provided skin coverage to the anterior ear and conchal bowl.

## 2. Case Report 

**Figure 1:**
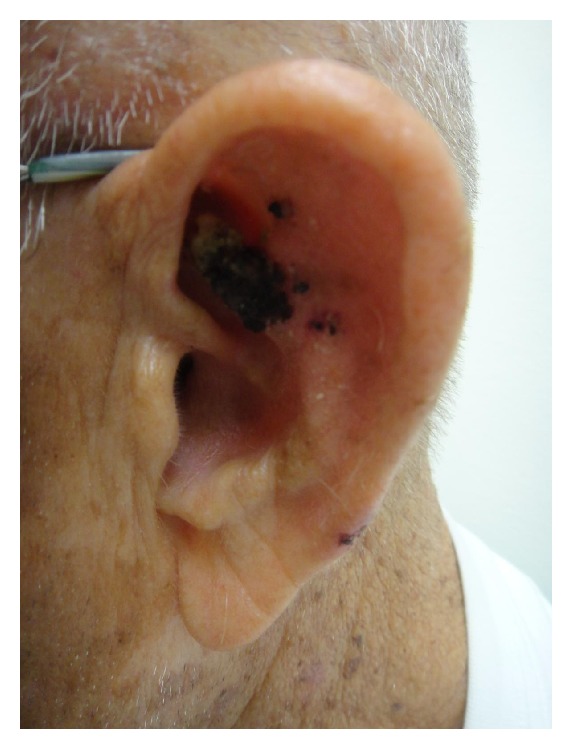
The preoperative photo of the left ear with squamous cell carcinoma on the antihelix.

**Figure 2:**
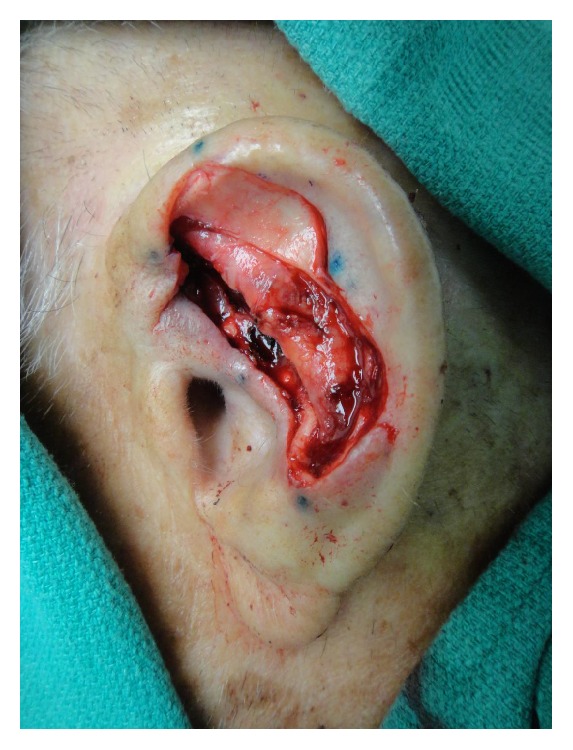
The final Mohs defect after two stages, measuring 4.6 × 2.4 cm.

**Figure 3:**
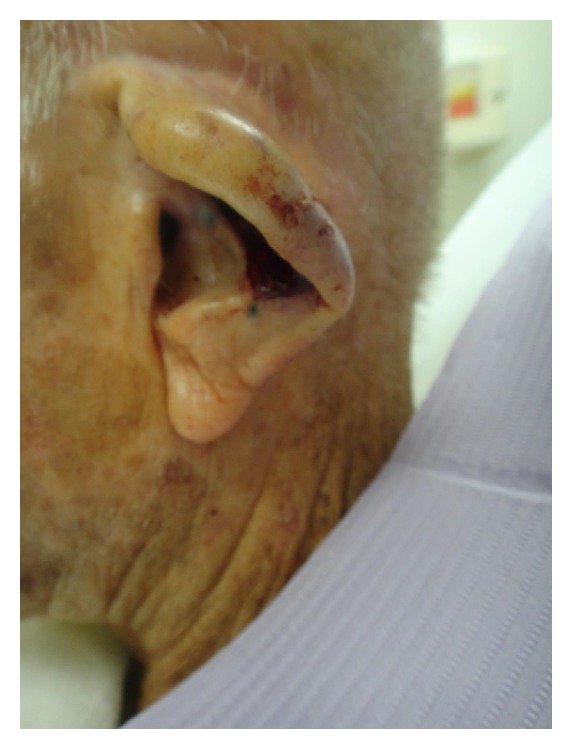
Lop-ear deformity.

An 84-year-old man underwent Mohs micrographic surgery for a primary squamous cell carcinoma on the left antihelix (Figure 1). At the time of the surgery, he was on anticoagulation therapy consisting of aspirin 81 mg po daily due to a history of myocardial infarction. He was given ciprofloxacin 500 mg by mouth twice daily starting two days preoperatively and continuing for a total of 14 days as surgical prophylaxis. Oncologic resection was complete after two stages, and the final defect measured 4.6 × 2.4 cm, involving the antihelix, scapha, underlying cartilage, and a small portion of the helix (Figure 2). The superior ear was structurally compromised due to the loss of cartilage, causing the ear to fold anteriorly (lop-ear deformity) (Figure 3). Retroauricular skin was intact, but there was exposed cartilage within the conchal bowl and at the scaphoid fossa.

**Figure 4:**
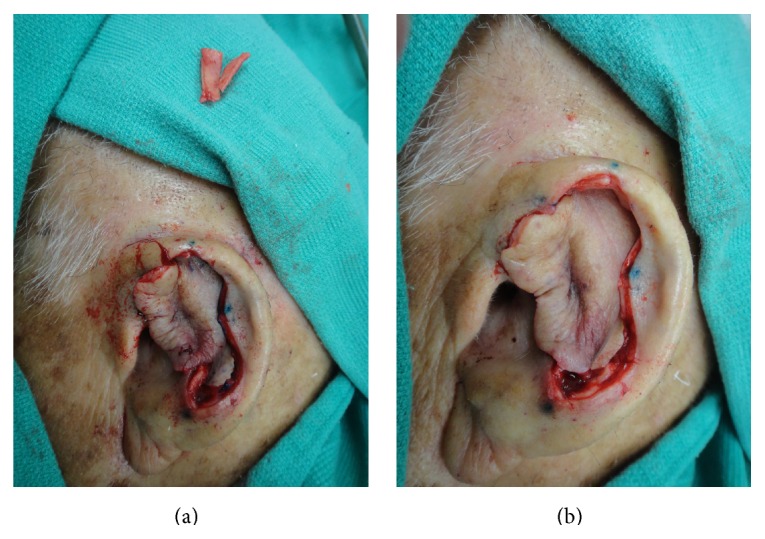
A 1.5 cm^2^ cartilage graft was harvested from scaphoid fossa and attached to the antihelix region with quilting sutures. The postauricular skin was folded forward through the cartilage window to cover the cartilage graft.

**Figure 5:**
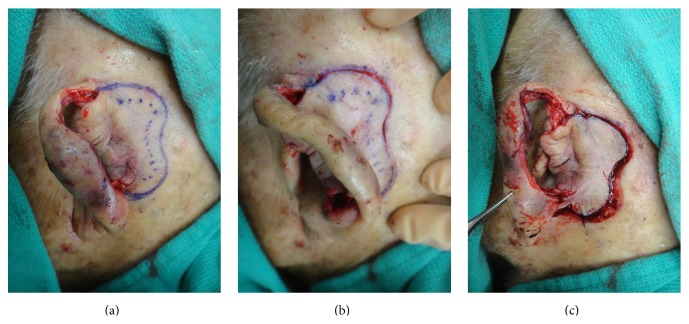
The defect served as a stencil on the postauricular skin to create the island, which was incised and undermined at the edges to allow for elevation.

Due to the size of the defect, limited laxity in surrounding tissues, and loss of structural integrity of the ear cartilage, primary closure and skin grafting were deemed to be suboptimal wound management strategies. Instead, a 1.5 cm^2^ cartilage graft was harvested from the exposed cartilage in the scaphoid fossa, sculpted, and sutured to the adjacent antihelix region with 4-0 poliglecaprone 25 suture, serving to recreate the crura of the antihelix (Figure 4(a)). The skin on the posterior auricle was then incised just below the helical rim, creating a full-thickness defect in the central ear. Skin elevation stopped at the auriculomastoid groove, leaving the skin based on a central subcutaneous pedicle, and the flap was folded anteriorly to cover the cartilage graft and then inset with 4-0 poliglecaprone 25 sutures tacking the flap dermis to the deeper tissues (Figure 4(b)). The full-thickness defect then served as the stencil template for the island portion of the revolving door flap (Figure 5(a)). The designed island was incised with 5 mm of undermining at the edges (Figure 5(b)) and then pulled through the cartilage window anteriorly (Figure 5(c)), thereby creating the revolving door. The flap was then inset into the ear defect anteriorly using 4-0 polypropylene suture. The wound edge on the posterior ear was similarly sutured to the incised skin edge on the postauricular skin, thereby pinning the helix to the mastoid region and correcting the lop-ear deformity. A tie-over bolster was made using fine mesh gauze impregnated with 3% bismuth tribromophenate in a petrolatum blend filled with sterile gauze fluffs and was affixed to the wound with 3-0 silk sutures. Patient was instructed to apply mupirocin 2% ointment to the postauricular suture line daily.

**Figure 6:**
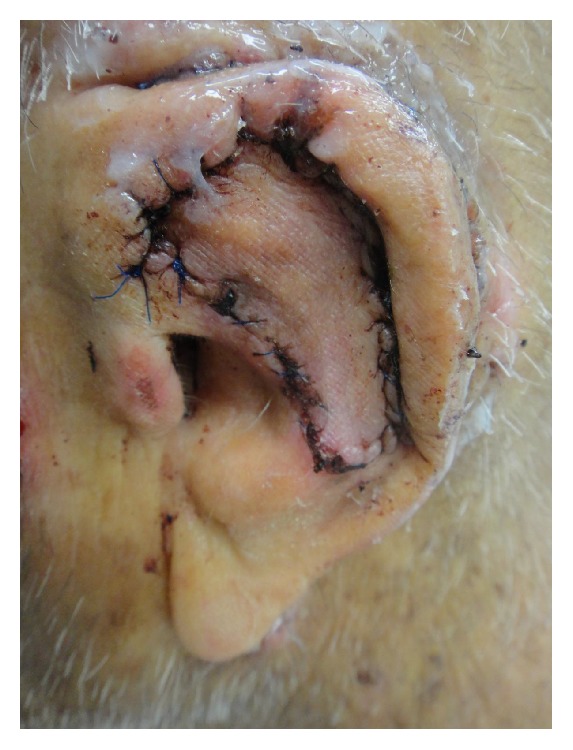
The flap was inset into the defect and sutured into place.

**Figure 7:**
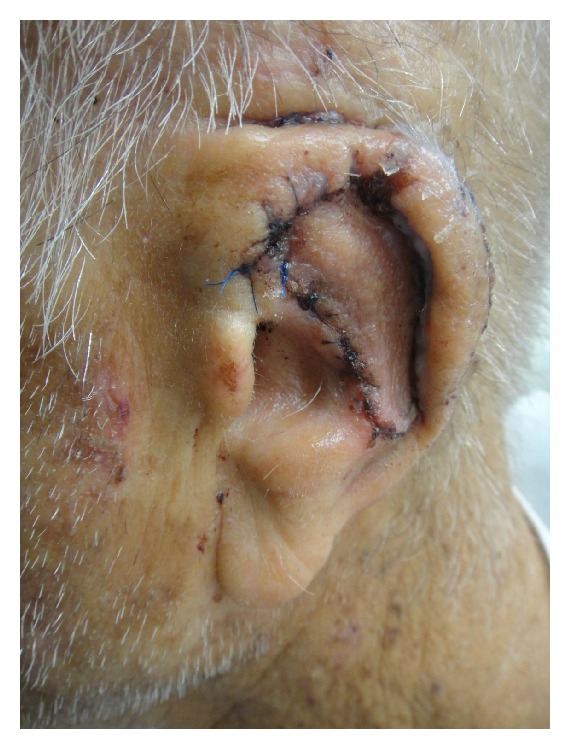
Two-week postoperative results.

**Figure 8:**
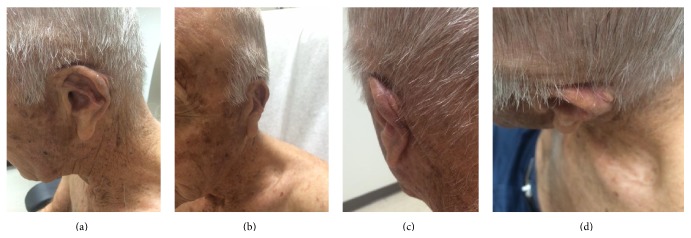
Three-month postoperative results.

The bolster was taken down at 48 hours due to postoperative bleeding from the suture line and concerns for a possible hematoma. Upon inspection, the flap appeared well-perfused with no signs of underlying fluid collection and no active bleeding. A new bolster was placed at that time and was removed 8 days postoperatively. The immediate closure and 2-week and 3-month postoperative results are shown in Figures 6, 7, and 8.

## 3. Discussion 

Though traditionally used for conchal bowl defects, postauricular revolving door (or trap door or flip-flop) island pedicle flaps can be used to repair large anterior ear defects that lack perichondrium and involve the helix, antihelix, and scapha [5, 6]. It was originally described by Masson in 1972 [7], with modifications by later authors serving to expand its indications to larger and more complex defects. While most commonly used for repair of Mohs defects after cancer resection, these flaps have also been successfully used in the repair of necrotic cartilage and antihelical skin caused by a second-degree burn [8]. This flap provides a varying amount of skin from the ipsilateral retroauricular and mastoid regions, depending on the size and position of the defect [4, 6], and has been described to cover defects as large as 6 × 6 cm [9]. The retroauricular skin has a rich blood supply, and island flaps utilizing this tissue minimize the risk of necrosis and hematoma formation [10]. The blood supply of this myocutaneous flap is the posterior auricular artery, which derives from the external carotid artery [9].

This flap has been described in previous literature as requiring more aggressive undermining of the postauricular skin, in order to prevent retroposition of the ear [3, 5]; up to 50% of the overall flap area may be undermined without affecting perfusion [11]. In this case, however, we felt that the patient would benefit from a superior cosmetic outcome if we used a modified, minimally elevated revolving door island flap in combination with a cartilage graft. This approach allowed us to simultaneously cover the exposed cartilage, recreate a portion of the normal contour, and correct the lop-ear deformity.

Advantages of this technique include color, texture, and thickness match, one-stage reconstruction, the ability to conceal the donor-site deformity, and results that are usually both functionally and aesthetically satisfactory for the patient [4, 6]. In addition to the postauricular area being relatively well concealed, adult patients typically have sufficient tissue to allow primary closure at the donor site [9]. Dessy et al. reported a superior cosmetic outcome with the revolving door flap compared to full-thickness skin grafts for wider skin tumor excisions of the auricular conchal defects among 40 skin cancer patients. Papadopoulos et al. describe a similar technique reconstructing the antihelix and concha with the postauricular island flap with excellent or adequate aesthetic outcomes in 74% and 24% of patients, respectively.

Disadvantages of this technique include pinning of the ear to the head, as with our patient, in addition to the surgical risks of necrosis, chondritis, infection, and the possible need for a postauricular drain (none of which occurred in this patient) [9]. Hematoma can also threaten the repair, and risk of hematoma is increased in patients on anticoagulation therapy, as in our patient. Meticulous hemostasis and flap immobilization with bolsters can minimize this risk. The degree to which pinning the ear to the head results in asymmetry and impacts the aesthetic outcome varies with how much an individual patient's ear naturally protrudes [3]. Other theoretical drawbacks include limited flap mobility due to the length of the pedicle and insufficient vascular supply in the event of parotid or mastoid surgery or external carotid artery ligation [9].

Additionally, we report an uncommon method of incorporating a cartilage graft underneath the flap to help recreate the ear's contour. Cartilage autografts such as the ones we used have been found to be superior to synthetic biomaterials or cadaveric grafts due to a lower risk of immunogenic rejections that cause inflammation and eventual graft failure. If available, as in this patient, the antihelix is the preferred harvest site for cartilage that will subsequently be used to enhance structural support of the auricle [12]. Use of a bolster or closed-suction drain after cartilage grafting, though cumbersome, is commonly used to maximize close adherence of flap to graft, minimize hematoma formation, and maximize aesthetic outcome. As with any reconstructive technique, proper planning and soft tissue management are imperative to minimize complications and further anatomical disfigurement.
